# A Bibliometric Analysis of Research on Bacterial Persisters

**DOI:** 10.1155/2023/4302914

**Published:** 2023-01-06

**Authors:** Yuan Ju, Fang Zhang, Pingjing Yu, Yu Zhang, Ping Zhao, Ping Xu, Luwei Sun, Yongqing Bao, Haiyue Long

**Affiliations:** ^1^Sichuan University Library, Sichuan University, Chengdu, China; ^2^Department of Pharmacy, The Air Force Hospital of Western Theater Command, Chengdu, China

## Abstract

**Background:**

In the past two decades, the surge of research on bacterial persisters has been inspired as increasingly concerning about the frequent failure of antibiotics treatment. This study was aimed at presenting a bibliometric and visualized analysis of relative publications on bacterial persisters, which offered insights into the development and research trends of this field.

**Methods:**

The Web of Science Core Collection and Ovid MEDLINE databases were utilized to retrieve relevant publications on bacterial persisters from 2001 to 2021. After manual selection, data including titles, authors, journals, author keywords, addresses, the number of citations, and publication years were subsequently extracted. The data analysis and visual mapping were conducted with Excel, SPSS, R studio, and VOSviewer.

**Results:**

In this study, 1,903 relevant publications on bacterial persisters were included. During 2001-2021, there was an exponential growth in the quantity of publications. It was found that these studies were conducted by 7,182 authors from 74 different countries. The USA led the scientific production with the highest total number of publications (859) and citation frequency (52,022). The *Antimicrobial Agents and Chemotherapy* was the most influential journal with 113 relevant publications. The cooccurrence analysis revealed that studies on bacterial persisters focused on four aspects: “the role of persisters in biofilms,” “clinical persistent infection,” “anti-persister treatment,” and “mechanism of persister formation.”

**Conclusion:**

In the past two decades, the global field of bacterial persisters has significantly increased. The USA was the leading country in this field. Mechanistic studies continued to be the future hotspots, which may be helpful to adopt new strategies against persisters and solve the problem of chronic infection in the clinic.

## 1. Introduction

The frequent failure of conventional antibiotic treatment has become a serious medical crisis worldwide, which increased mortality, time of hospitalization, and healthcare costs [1]. Several factors in host and pathogen contribute to the ineffectiveness of antibiotic treatment, such as immunosuppression, immune escape, and antibiotic resistance [2]. Moreover, the role of persister formation in antibiotic treatment failure is steadily gaining recognition [3, 4]. Persisters are a small population of bacteria (typically less than 1%) that can enter into metabolically dormant and slow-growing states after exposure to high-dose antibiotics [5]. In contrast to antibiotic-resistant bacteria with acquiring mutations, persisters are phenotypically heterogeneous variants with antibiotic tolerance and can resume growing once the antibiotics are removed [6, 7]. Meanwhile, persisters have been shown to accelerate the emergence of antibiotic resistance [7, 8]. In addition, persisters are related to the formation of biofilms, and the presence of persisters in biofilm is largely responsible for the resistance of biofilms [9, 10]. Biofilms are communities of bacteria where cells are in highly hydrated extracellular matrix with proteins, polysaccharides, lipids, and extracellular DNA [11]. Infections related to biofilms are difficult to be eliminated, and biofilm-derived persister formation is strongly implicated in the relapse and recalcitrant nature of recurrent chronic infections [4, 10]. Nowadays, several strategies are being developed to identify novel compounds that can directly kill persisters or eradicate persisters in biofilms by combing them with antibiotics [12, 13].

Bibliometric analysis is a powerful tool, which utilizes mathematical and statistical methods to assess the change in the number of publications, countries, authors, keywords, etc. over time [14]. It can evaluate the quality of publications, collaboration patterns, and research trends and compare academic impact or research progress among different countries, institutions, or authors [15, 16]. Moreover, The VOSviewer is an efficient program to construct bibliometric maps and reveal the characteristics of research at the microscopic and quantitative levels [17]. Bibliometric analysis has been applied to various fields, including medicine, psychology, biology, and ecology [18–21]. Also, several bibliometric studies were focusing on pathogenic bacteria and biofilm [22, 23].

As the field of bacterial persisters has drawn more attention, it is vital to characterize the evolution of this field in detail. Therefore, this study was aimed at tracking the global development status and analyzing the research trends of bacterial persisters through a bibliometric analysis of related publications within the past 20 years. According to the data from the Web of Science Core Collection (WoSCC) and Ovid MEDLINE databases, the contributions of individual countries, institutions, authors, and journals were analyzed to give insights into the field of interests, academic impacts, and collaboration patterns. Besides, the visualized map helped to obtain the research trends and hotspots in the field of bacterial persisters. The results of this study might provide a theoretical basis and new ideas for further in-depth research on bacterial persisters.

## 2. Methods

### 2.1. Data Source

The MeSH terms from PubMed and Emtree terms from Embase were used. The truncated search terms were used to increase the scope of the search for words that have singular and plural forms or variant endings. In addition, the Boolean operators “AND,” “OR,” and “NEAR” were utilized to construct the search strategy. The WoSCC and Ovid MEDLINE databases were utilized to retrieve all relevant literature in this bibliometric analysis. The literature retrieval was performed on September 6, 2022 to identify studies published between January 1, 2001 and December 30, 2021. The search strategies in WoSCC and Ovid MEDLINE are listed in Table S1.

### 2.2. Data Collection

Consequently, a total of 9,317 studies were retrieved. Endnote X9 was utilized to remove the duplicated references. Then, three authors independently assessed the titles or abstracts and excluded irrelevant studies to ensure that the studies focused on bacterial persister. Studies that only mentioned persister in passing were excluded. Any discrepancies were resolved by consensus-based discussion of full texts or corroborated by another author. As a result, 1,903 valid publications were included, and the following information was extracted from each article: title, authors, journal, publication year, document type, keywords, addresses, subject category, and the number of citations in the WoS database. The *h*-index was analyzed by the intrinsic functions of the WoS database, and the 2021 impact factors (IFs) of the journals were supplemented from the Journal Citation Reports (JCR, https://jcr.clarivate.com/).

### 2.3. Statistical Analysis

Microsoft Excel and IBM SPSS Statistics were utilized to count the frequency of descriptive characteristics, like countries, authors, and the number of citations. The annual growth rate (AGR) was calculated using the equation suggested by Gracio et al. [24]. The compound annual growth rate (CAGR) was calculated using the equation of Choi et al. [25]. The relative growth rate (RGR) and doubling time (DT) were calculated on the equations utilized by Shukla and Verma [26]. The degree of collaboration (DC) in different years was calculated using the following formula [27]: DC = Nm/(Nm + Ns), where Nm was the number of multi-author publications and Ns was the number of single-author publications. The Spearman rank was utilized to analyze the correlation between variables. Graphs were made by R Studio and Microsoft Excel. VOSviewer 1.6.17 (https://www.vosviewer.com/) [17, 28] was utilized to construct cooccurrence networks of countries, institutions, authors, and keywords.

## 3. Results

### 3.1. Trends of Global Publication and Citations

**Figure 1:**
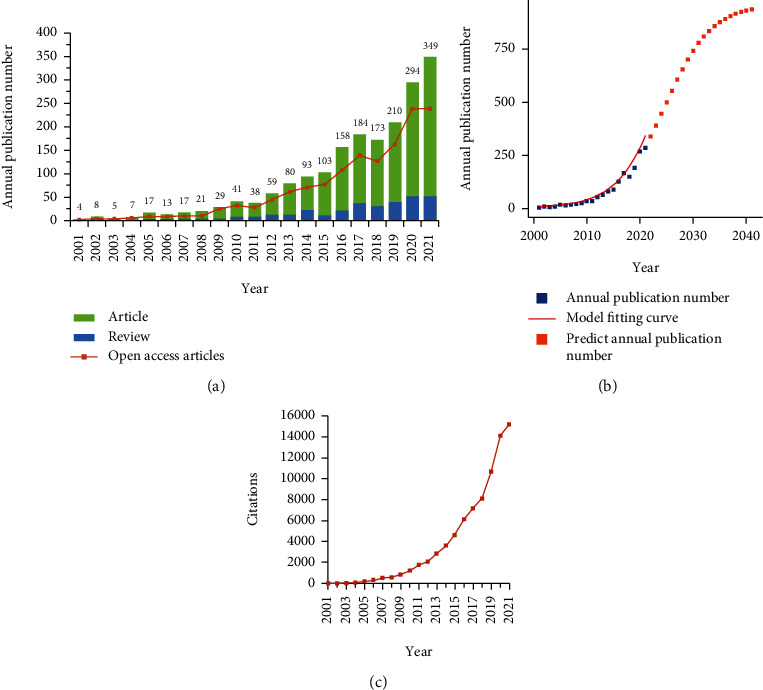
Global publishing trends on bacterial persisters (2001-2021). (a) The total number of publications, the number of open-access articles, proportion of articles, and proportion of reviews on bacterial persisters per year. (b) Model fitting curves related to growth trends in the number of worldwide publications. (c) Sum of citations received per year.

In this research, there were 1,903 records related to bacterial persisters from 2001 to 2021.  Figure 1(a) shows the distribution of the publications by year. Accordingly, it was found that global publications on bacterial persisters exhibited a strong trend for exponential growth, from 4 publications in 2001 to 349 publications in 2021. Moreover, open-access articles occupied a dominant position in published papers every year. The number of reviews progressively increased from 2001 to 2021, and it persistently remained at lower than 25%. In addition, the logistic regression model was used to build the time curve of the annual publication number and predict future publication trends (Figure 1(b)). The results indicated that the field of bacterial persisters is currently in the early stage of rapid growth, and the number of publications will grow rapidly for the next decade.

The quantitative characteristics of the publications on bacterial persisters could be divided into three stages. In the first stage (2001-2007), the study on bacterial persisters was at an initial stage. At this stage, a total of 71 papers were published accounting for only 3.73% of the total publications (Table 1). Moreover, the annual publications were no more than 20 with CAGR of 27.27%. At this stage, the maximum AGR (142.86%) was recorded for 2005, and the minimum AGR (-37.50%) was recorded for 2003. The RGR decreased from 1.10 in 2001 to 0.27 in 2007, and the DT increased from 0.63 years in 2001 to 2.53 years in 2007. Although few studies were performed at the initial stage, the preliminary exploration of bacterial persisters offered a theoretical foundation for further research. The second stage (2008-2015) was regarded as a rising stage with AGR of about 30% every year, except for the particular year of 2011. There was an average of 58 papers published every year, and the total number of 464 publications accounted for 24.38% of this research. In addition, the CAGR of this stage arrived at 25.50%, the RGR remained between 0.21 and 0.29, and the DT ranged from 2.37 years to 3.29 years. With the continuous deepening of the research on bacterial persister, the third stage (2016-2021) was a boom stage with an average of 228 publications every year and CAGR of 17.17%. Moreover, the RGR ranged from 0.18 to 0.26, and the value of DT increased to 3.42 in 2021. These results showed that more and more attention has been paid to the study of bacterial persisters around the world.

In terms of citations, Figure 1(c) shows that the number of citations per year generally increased in the past two decades. The Spearman rank test revealed a positive and statistically significant correlation between the length of published time and frequency of citations (*r* = 0.991, *P* < 0.001), as well as between the annual publications and annual citations (*r* = 0.991, *P* < 0.001). Furthermore, the most cited article (1,847 citations) was published in 2004 by Professor Nathalie Q. Balaban, describing the persistence switch between normally growing cells and persister cells having reduced growth rates [5].

### 3.2. Distribution and Cooperation of Countries

**Figure 2:**
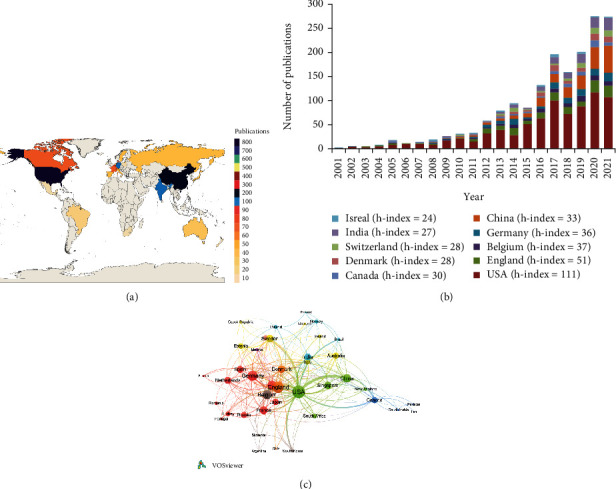
Spatial distribution of publications on bacterial persisters (2001-2021). (a) Geographical distribution of publications on bacterial persisters. (b) The annual distribution of bacterial persister research from top 10 influential countries between 2001 and 2021. (c) Cooperation network of the productive countries with a minimum of 20 publications. The node size represented the number of publications, and the thickness of the links represented the close degree between countries.

The papers related to bacterial persisters originated from 74 countries. The geographical distribution by the number of publications is shown in Figure 2(a). Most applied studies were performed in the USA with 859 papers (45.16%) and China with 231 papers (12.14%), followed by England (188 papers, 9.88%), India (133 papers, 6.99%), and Germany (118 papers, 6.20%). Other countries posted no more than 100 papers. This indicated that USA and China attached great importance to the field of bacterial persisters. In the WoSCC database, the USA had the highest number of citations (52,022) with the *h*-index of 111, followed by England (9,758, *h*-index of 51), Belgium (4,646, *h*-index of 37), Germany (4,411, *h*-index of 36), China (4,596, *h*-index of 33), and Canada (4,759, *h*-index of 30). In addition, Gambia showed the highest average frequency of citations with 259 citation counts for only one publication, describing nonreplicating persisters in tuberculous sputum [29]. Israel ranked second with 172 average citation counts per paper, followed by Estonia (92), Jordan (89), and United Arab Emirates (89).

Figure 2(b) shows the annual distribution of studies on bacterial persisters in the top 10 influential countries. The USA dominated in terms of the number of publications from 2001 to 2021, and England remained at second from 2001 to 2014. After 2015, the number of publications from China or India has been increasing and persistently ranked at around the second and third place. These results suggested a great potential for the development of bacterial persister research in Asia.

International cooperation was found between 68 countries.  Figure 2(c) shows the network of the productive countries with publications greater than 5 and demonstrates the collaborative ties among countries. Four countries were presented in nine different clusters with different colors, and there was an intensive collaboration among the countries within the same cluster. The size of the node reflected the number of publications for each country, and the thickness of the line was correlated to the strength of research collaboration. Among them, the USA stood in the center of the network with links (*L*) = 35 and total link strength (TLS) = 302. The thickest line is connected to China, and this represented that the USA had the strongest cooperation with China in the field of bacterial persisters. This is followed by England which had *L* = 30 and TLS = 106; Germany, *L* = 26 and TLS = 61; and Belgium, *L* = 23 and TLS = 40. Furthermore, Australia, Canada, Iran, Pakistan, and Saudi Arabia were related to the same cluster in this network. Israel and Mexico were in a separate cluster, respectively.

### 3.3. Distribution and Cooperation of Institutions

Through analyzing the distributions and cooperation of research institutions, key institutions with large publications and high impact on bacterial persisters could be identified. A total of 1,512 institutions have participated in the research of bacterial persisters. The top 20 influential institutions according to *h*-index are listed in Table 2, the top 20 productive institutions according to the number of publications are listed in Table S2, and the top 20 cited institutions are listed in Table S3. Accordingly, Northeastern University was the most influential institution in the field of bacterial persisters as it had the highest *h*-index (41) and number of citations (12,609); meanwhile, it ranked third in the list of top 20 productive institutions with 55 publications. Johns Hopkins University was the institution with the most number of publications (69), but the citation count of Johns Hopkins University was much less with a total of 2,409 citations and *h*-index of 28. It was worth noting that Rockefeller University published only 9 papers but ranked fifth in the list of top 20 cited institutions with 3,041 citations. This indicated that Rockefeller University caused a considerable impact. In terms of the top 20 influential institutions, there were 12 institutions from the USA with an *h*-index ranging from 41 to 16. Moreover, there were 2 institutions in China with an *h*-index of 14. In terms of the top 20 prolific institutions, there were 11 institutions from the USA with a total of 404 publications. Similarly, 2 productive institutions were from China and had 51 publications. In addition, the distribution of the top 20 influential institutions demonstrated that studies on bacterial persisters were mainly concentrated on universities (17) and research institutions (2). The number of publications for institutions showed a not very strong correlation with citations (*r* = 0.606, *P* < 0.001).

**Figure 3:**
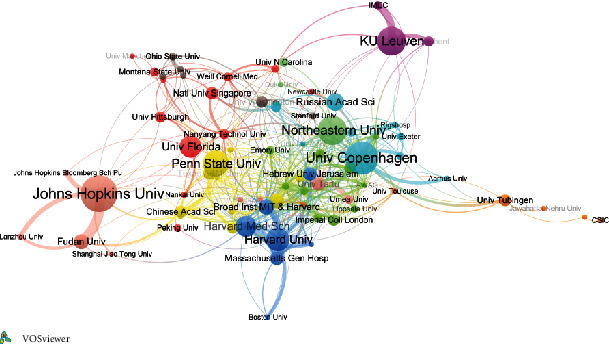
Coauthorship network of the productive authors with a minimum of 5 publications. The node size represented the total links of each institution, and the thickness of the links represented the close degree between institutions.

The cooperation network of institutions with a minimum of 10 publications was constructed. Syracuse University, University of Calgary, Indian Institute of Science, Ilam University of Medilam Sciences, and Sungkyunkwan University were excepted as they were “remote” institutions without connecting to other institutions. The size of the node represented the number of links for each institution. 77 connected institutions were presented in 11 clusters with 436 links. As shown in Figure 3, the top 5 institutions with the largest TLS were Harvard University (TLS = 142), Broad Institute of MIT and Harvard (TLS = 141), University of Copenhagen (TLS = 133), Harvard Medical School (TLS = 132), and Massachusetts General Hospital (TLS = 112). Meanwhile, these institutions are connected and some of them belonged to the same cluster; this indicated that their cooperation was very intensive.

### 3.4. Distribution and Cooperation of Authors

**Figure 4:**
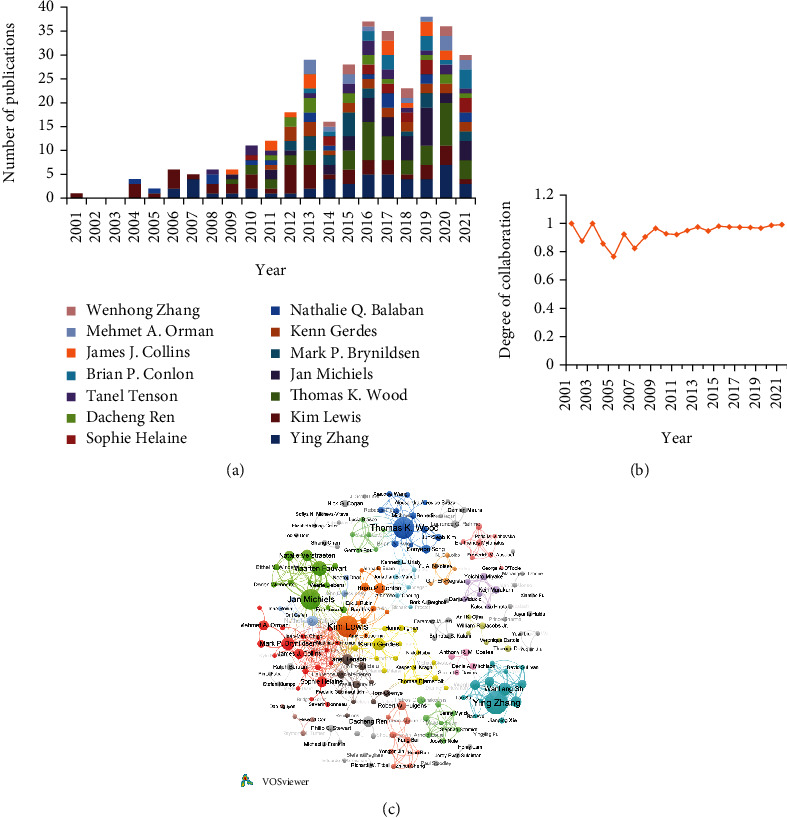
Authors in the field of bacterial persisters (2001-2021). (a) Average output of the corresponding authors in the list of top 19 productive authors on bacterial persister-related studies between 2001 and 2021. (b) Trend of degree of collaboration (DC) of publications. (c) Coauthorship network of the productive authors with a minimum of 5 publications. The node size represented the total links of each institution/author, and the thickness of the links represented the close degree between institutions/authors.

Through analyzing the characterization of the authors' cooperation network, it identified the core group of authors and social connection among researchers on bacterial persisters. 7,182 authors were involved in the publications in the field of bacterial persisters. A total of 19 authors published at least 15 papers on bacterial persisters (Table 3). Ying Zhang from Johns Hopkins University was the most productive author with 54 publications totalizing 1,712 citations, followed by Thomas K. Wood from Pennsylvania State University with 49 publications, and Kim Lewis from Northeastern University with 48 publications. Among all authors, Kim Lewis was the most influential author with a total of 12,295 citations, followed by Nathalie Q. Balaban from The Hebrew University of Jerusalem with a total of 5,075 citations. James J. Collins from Broad Institute of MIT and Harvard ranked third with 3,470 citations. Among the top 19 productive authors, 14 scholars published papers as corresponding authors and the output of these corresponding authors between 2001 and 2021 is presented in Figure 4(a).

Authors in the field of bacterial persisters presented a high trend for collaborations with DC ranging from 0.76 to 1.00 (Figure 4(b)). From 2001 to 2005, the degree of collaboration between authors declined, reaching its bottom in 2005 (0.76). This might be because there were fewer papers published and single-authored papers showed a great impact on DC. Since 2007, the desire for coauthorship was increasing and the value of DC maintained above 0.9. Among the investigated publications, the multi-authored papers (96.90%) dominated over the single-authored papers (3.10%), and the mean value of DC from 2001 to 2021 was 0.94.

The network of authors with 5 or more papers was considered, and 205 authors are shown in Figure 4(c). These authors were divided into 42 clusters with 583 links. The size of the node represented the total link strength of each author. Jan Michiels and Thomas K. Wood accounted for the largest cooperation with TLS = 100 and TLS = 92, respectively. As shown in Figure 4(c), closely connected cooperative groups of authors from different institutions have been formed, including the connection between Kim Lewis from Northeastern University, Nathalie Q. Balaban from The Hebrew University of Jerusalem, Jan Michiels from Catholic University of Leuven, Brian P. Conlon from University of North Carolina, and Tanel Tenson from University of Tartu. In addition, Thomas K. Wood from Pennsylvania State University, Maria Tomas from Universidade da Coruna, and Kenneth L. Urish from University of Pittsburgh have also formed a close cooperative group.

### 3.5. Distribution of Journals

There were 451 journals that participated in the research on bacterial persisters, and the 2021 impact facts (IFs) of these journals ranged from 0.209 (*Southeast Asian Journal of Tropical Medicine and Public Health*) to 112.288 (*Nature Reviews Drug Discovery*). Moreover, there were 41 journals publishing at least 10 papers concerning bacterial persisters. The top 10 popular journals with the largest number of publications or highest number of citations were listed in Table 4 and Table S4. For the number of publications, the *Frontiers in Microbiology* ranked first with 115 publications, followed by *Antimicrobial Agents and Chemotherapy* (113), and *Journal of Bacteriology* (67). The 2021 IFs of these journals are 6.064, 5.938, and 3.476, respectively. In addition, *Antimicrobial Agents and Chemotherapy* and *Journal of Bacteriology* obtained a significant frequency of citations (5,725 and 5,121, respectively). Moreover, *Science* and *International Journal of Antimicrobial Agents* published less than or equal to 10 papers but possessed a large frequency of citations (5,089 and 2,128, respectively). For journals, the Spearman rank test indicated that there was a low correlation (*r* = 0.698, *P* < 0.001) between the number of publications and citation counts. Within journals that had 2021 IFs, the number of publications (*r* = 0.151, *P* = 0.003) and citation counts (*r* = 0.372, *P* < 0.001) both showed weak relationships with 2021 IFs.

The *h*-indexes of journals could be used to evaluate the impact of journals. Among the top 10 popular journals, *Antimicrobial Agents and Chemotherapy* had the highest *h*-index of 41, and *Journal of Bacteriology* ranked second with an *h*-index of 34. *Frontiers in Microbiology* and *mBio* both had an *h*-index of 28, but the number of publications had a double difference. For the top 10 popular journals, the Spearman rank test indicated that the number of publications (*r* = 0.802, *P* = 0.05) and citations (*r* = 0.979, *P* < 0.001) was closely related to the *h*-indexes of journals. However, the 2021 IFs were not correlated to the *h*-indexes of journals (*r* = 0.226, *P* = 0.559).

### 3.6. Cooccurrence of Keywords

**Figure 5:**
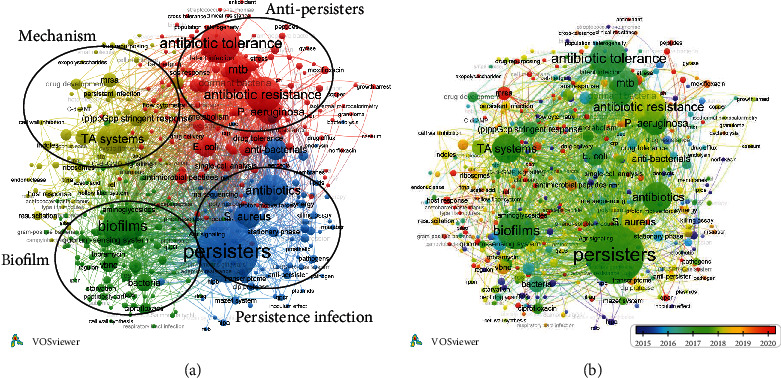
The cooccurrence network of author keywords occurred more than 2 times. (a) Mapping of keywords in the research. The keywords were divided into 4 groups as follows: “persisters in biofilms,” “clinical persistent infections,” “anti-persister treatment,” and “mechanism of persister formation.” The size of the node represented the frequency of occurrence. (b) Distribution of keywords according to the average appearance time. Keywords in purple appeared earlier than those in blue, green, and yellow, and keywords in red appeared the latest.

The keywords were identified as words that have been used in author keywords. After cleaning, screening, and inference, the network offered 439 keywords that appeared more than 2 times (Figure 5(a)). The keyword analysis revealed that “persisters” (465 occurrences), “biofilms” (189 occurrences), “antibiotic tolerance” (186 occurrences), “antibiotic resistance” (150 occurrences), “antibiotics” (117 occurrences), “TA systems” (111 occurrences), “Mtb” (86 occurrences), “S. aureus” (76 occurrences), “P. aeruginosa” (74 occurrences), and “E. coli” (46 occurrences) were more frequent. The clusters of keywords identified the primary groups of subjects, and the size of the node represented the occurrences of the keywords. From the results of cooccurrence analysis, studies on bacterial persisters were mainly concentrated in 4 aspects: (1) the role of persisters in biofilm, (2) clinical persistent infections, (3) anti-persister treatment, and (4) mechanism of persister formation. These encompassed the majority of published research on bacterial persisters.

These keywords were then divided by specific colors based on the average time they appeared in all publications (Figure 5(b)). As the global publishing on bacterial persisters boomed since 2015, the average publication time of these keywords mainly distributed from 2015 to 2020. The blue end of the spectrum indicated that a keyword appeared in earlier studies (2015), whereas the red-colored keywords appeared in recent studies (2020). The keywords in groups of “the role of persisters in biofilm” and “mechanism of persister formation” were the major area in 2018-2020. Meanwhile, research focused on “magnetite nanoparticles,” “prosthetic joint infection,” “protein aggregation,” and “SAAP-148” appeared to be keywords attracting more attention in 2021, and they might be extensively concerned in the future.

### 3.7. Distribution of Significant Research Areas

**Figure 6:**
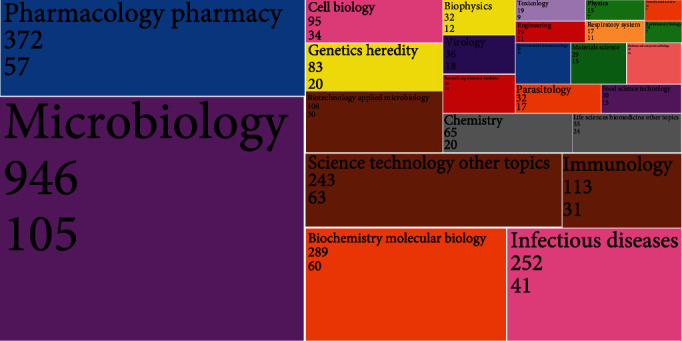
Word tree-map of research areas in bacterial persister theses (2001-2021). The number of publications, total citation counts, and *h*-index were included for the research areas.

The publications on bacterial persisters could be concentrated on 67 areas according to the research areas in the WoS database. The areas with more than 10 publications are shown in Figure 6. Among them, “Microbiology” was the most frequent area with 946 publications (49.71%). There followed six research areas that reached more than 100 publications, including “Pharmacology Pharmacy,” “Biochemistry Molecular Biology,” “Infectious Diseases,” “Science Technology Other Topics,” “Immunology,” and “Biotechnology Applied Microbiology.” Besides, the areas including “Genetics Heredity,” “Chemistry,” “Virology,” “Food Science Technology,” “Materials Science,” and “Mathematical Computational Biology” had publications related to bacterial persisters. Consistent with the number of publications, the area of “Microbiology” had the highest total citations (47,090) and *h*-index (105). In terms of citations, “Science Technology Other Topics” ranked second with total citations of 17,307 and *h*-index of 63, followed by “Pharmacology Pharmacy” with 14,241 citations and *h*-index of 57, “Biochemistry Molecular Biology” with 13,854 citations and *h*-index of 60, and “Infectious Diseases” with 7,498 citations and *h*-index of 41. This indicated that there were more citations of multidisciplinary research areas.

## 4. Discussion

In this study, we explored the bibliometric characteristics and visualized analyses of the field of bacterial persister research using VOSviewer. The results might provide references and suggestions for further research on bacterial persisters.

### 4.1. Publishing Trends

During 2001-2021, 1,903 relevant publications were produced in the field of bacterial persisters. In 2001, the field of bacterial persisters has not attracted much attention with only 4 publications. At this time, Spoering and Lewis proposed that the presence of persisters in biofilm played an important role in antibiotic tolerance of biofilm [9]. Since then, there was an exponential increase in publication output, and the number of publications was expected to grow rapidly over the next decade. Meanwhile, the citation frequency had generally increased over time with the development of research on bacterial persisters. This growth is mainly due to the clinical importance of persisters in biofilm tolerance and chronic infections, which had kindled increasing research interest in understanding the mechanisms of persister formation and developing anti-persister strategies to eliminate persistent infections.

### 4.2. Countries

The USA is the world leader in the field of bacterial persisters, which had the largest collection of publications (859) and the highest total citation frequency (52,022) and *h*-index (111). The USA stood at the center of the cooperation networks countries and had more link strength with other countries (Figure 2(b)). More than half of the top 20 influential institutions (12 institutions) were located in the USA (Table 2). These trends indicated that the USA had a strong economic foundation and better research bases to support the in-depth study on bacterial persisters. Meanwhile, publications from the USA had high academic impacts and strong collaborations worldwide in this field. On the other hand, the problem of bacterial persisters attracted attention in several European countries, like England, Germany, Belgium, Denmark, Switzerland, and Sweden. These countries have seen increasing publication numbers, impacts, and collaborations with other countries. However, most of the international collaborations were between countries in European and North America. Asian countries, including China, India, South Korea, and Japan, showed weak cooperative relations. More extensive international cooperation should be carried out in the future.

Over the past two decades, the number of publications on bacterial persisters arising from China and India has increased rapidly, as the total number of publications in China has ranked second among countries worldwide. However, China stood at the fifth place in *h*-index and the sixth place in the total citation frequency. There were several reasons that led to this distance between the quantity and the academic impact of publications. Firstly, publications on bacterial persisters from China were mainly published after 2015. Simultaneously, citations usually have a delay of at least 2 years, and publications by Asian countries are more difficult to receive prompt citations than by western countries [30]. Secondly, the academic evaluation system in China mainly focused on the quantity of publications [31]. This indicated that Chinese academics should gradually improve the quality of publications in this field and pay more attention to increasing the citation frequency of publications in the future.

### 4.3. Institutions and Authors

Unsurprisingly, the highest academic output of institutions was closely related to the top contributing countries. In terms of *h*-index or the number of publications, 60% or 55% institutions of the top 20 influential institutions or productive institutions originated from the USA, respectively (Table 2 and Table S2). The Johns Hopkins University (69 publications) was the most productive institution globally; meanwhile, Northeastern University was the institution whose publications caused the greatest academic impact (*h*-index of 41). Moreover, authors from the USA and Europe demonstrated a domain position, and most of the productive corresponding authors continuously published between 2011 and 2021 (Figure 4(a)). Ying Zhang from the Johns Hopkins University was the most productive author with 54 papers between 2006 and 2021, mainly involving the mechanism of persister formation in *E. coli*, *S. aureus*, or *M. tuberculosis*, as well as the identification of compounds against persisters [32–34]. Otherwise, Kim Lewis from the Northeastern University has published several papers that have been cited more than 500 times [9, 35–37]. Kim Lewis first reported the presence of persisters in biofilm in 2001. Subsequently, Kim Lewis, in collaboration with Iris Keren, Amy L. Spoering, Marin Vulić, and others, conducted extensive studies on the isolation of persisters, the characteristics of persisters, the mechanism of persister formation, and the development of antipersister compounds [12, 38–40]. These publications laid foundation for the research of bacterial persisters.

From the cooperation network map of institutions and authors (Figures 3 and 4(c)), it could be found that there was relatively close cooperation between the productive institutions. Authors in the field of bacterial persisters had a high degree of collaboration with an average DC from 2001 to 2021 of 0.94. However, the cooperation among authors in different institutions was not close. Regarding the top 19 productive authors, only one complicated and cross-institution cooperative network had formed, involving cooperation between Kim Lewis from Northeastern University; Maarten Fauvart, Jan Michiels, Natalie Verstraeten, and Bram Van Den Bergh from Catholic University of Leuven; Mark P. Brynildsen from Princeton University; Nathalie Q. Balaban from Hebrew University of Jerusalem; Sophie Helaine from Imperial College London; Tanel Tenson from the University of Tartu; Brian P. Conlon from the University of North Carolina; James J. Collins from the Broad Institute of MIT and Harvard; and Mehmet A. Orman from the University of Houston. Ying Zhang from Johns Hopkins University and Thomas K. Wood from Pennsylvania State University separately formed a cooperative group. We speculate the reason for this phenomenon was that some authors might label more than one institution in the paper and authors tended to collaborate with specific research groups. Thus, it is necessary to strengthen exchanges and in-depth cooperation between author collaboration groups.

### 4.4. Journals

The number of publications and citation frequency of journals indirectly reflected the journal ranking in the field of bacterial persisters. The majority of papers were published in microbiology journals, such as *Frontiers in Microbiology*, *Antimicrobial Agents and Chemotherapy*, *Journal of Bacteriology*, *mBio*, and *Journal of Antimicrobial Chemotherapy*. These journals were favored by academics in the field of bacterial persisters. Furthermore, *Antimicrobial Agents and Chemotherapy* was the most influential journal in the field of bacterial persisters with 5,325 citations and *h*-index of 41. This journal also ranked first in other bibliometric analyses, including in carbapenem resistance [41], antifungal triazole resistance [42], and antibiotic resistance of *Acinetobacter baumannii* [22]. This suggested its high reputation and authority in the field of microbiology. *Science* was ranked third in terms of citation frequency with only 10 publications (Table S4), which mainly published papers about crucial targets and mechanisms of bacterial persistence [43–45]. *Nature Reviews Drug Discovery* had the highest impact factor (112.288) and achieved the highest citation/publication (984) with only 1 paper. This paper reviewed strategies to establish platforms for identifying drugs against persisters [46]. In addition, 77 publications of the top 100 cited studies were open access. Open access is likely to accelerate dissemination of research findings and receive more citations [47, 48].

### 4.5. Trend Topics

The cooccurrence network of keywords severed as an important indicator to reflect the research hotspots and directions. Broadly speaking, the research directions in the field of bacterial persisters could be included into four aspects: the role of persisters in biofilm, clinically persistent infection, anti-persister treatment, and mechanism of persister formation. Several keywords lacked strong cooccurrence links with other keywords, like “ribosome hibernation,” “resuscitation promoting factors,” and “protein acetylation.” Ribosome hibernation was a process that bacteria polymerized 70S ribosomes into inactive 100S ribosomes [49]. In 2015, McKay and Portnoy reported that ribosome hibernation drove the development of antibiotic tolerance [49]. Then, Song and Wood proposed the ppGpp ribosome dimerization persister (PRDP) model, indicating that ppGpp induced the formation of persisters by regulating hibernation promoting factor- (Hpf-) mediated ribosome hibernation [50]. Studies on the topic of “resuscitation promoting factors” were performed between 2010 and 2020. Resuscitation promoting factors (RPFs) are muralytic enzymes in *M. tuberculosis* that stimulate the growth of persisters [51]. They have been widely used in the *in vitro* or *in vivo* models to detect the presence of Mtb persisters and shorten the treatment duration [51, 52]. Research on “protein acetylation” in the field of bacterial persisters started in 2016 when Cheverton et al. found that TacT acetylated tRNA to promote persister formation [53]. After that, other acetyltransferase toxins in the toxin-antitoxin system (TA system) were reported to acetylate aminoacyl-tRNA and Met-tRNA and regulate the formation of persisters [54–56]. Although these keywords were not mainstream research topics in this study, they showed great importance in understanding the mechanism of persister formation and were definitely worth further studying.

In this study, the clusters of keywords revealed that the research on bacterial persisters was mainly based on studies of *E. coli*, *P. aeruginosa*, *S. aureus*, and *M. tuberculosis* (Mtb). According to our bibliometric study, the appearances of “TA system,” “Agr quorum sensing (QS) system,” “(p)ppGpp stringent response,” “antipersister molecules,” “virulence factors,” and “oxidative stress” were more frequent. The activation of TA systems by (p)ppGpp has become a widely accepted model of persister formation [57]. The hipBA module was the first reported to have effect on the frequency of persistence, suggested by Moyed and Bertrand in 1983 [58]. Afterward, RelE toxin in the RelBE module was suggested to increase the formation of persisters by Keren et al. [36]. Other TA modules, like MqsR/MqsA, hicAB, and mazEF, also played major roles in the formation of persisters [59–62]. Furthermore, virulence factors were closely correlated with persister formation and biofilm development [63, 64]. Several regulators in QS system and synthetases in (p)ppGpp stringent response played important roles in phenotypic persistence through regulating multiple virulence factors [65–67]. Hence, identifying anti-persister molecules that interfered (p)ppGpp stringent response, TA system, and QS system has become a promising strategy to combat persisters [68–70]. Moreover, other antipersister molecules, including antimicrobial peptides which directly killed persisters and compounds that alleviated persistence into antibiotic-susceptible state or revitalized antibiotic efficacy, were developed to kill persisters [12, 71–73]. The environmental insults, like oxidative stress, also provoked the formation of bacterial persisters [74]. Under antibiotic treatment, reactive oxygen (ROS) was produced by Fenton reaction and then induced the oxidative stress response in bacteria [75, 76]. Subsequently, the increased expression of efflux pumps and secretion of indole could upregulate the SOS response [77]. Under these conditions, the SOS response induced persister formation by regulation of several toxins and SOS-dependent DNA repair [76, 78, 79]. Moreover, engineered bacteriophages and mesalamine were developed to kill persister by interfering with the oxidative stress in bacteria [80, 81].

The organization of keywords by average publication year provided insights into future trends in the field of bacterial persisters (Figure 5(b)). The keywords, which colored toward the red of the spectrum, mainly belonged to the “mechanism of persister formation” group. This indicated that mechanistic studies will gain further attention in this field. So far, research on the mechanism of the formation of persisters mainly focused on the effect of TA system, QS system, and (p)ppGpp stringent response [70]. However, the specific regulatory pathway of persister formation remains controversial. In addition, other factors, like ribosome hibernation and ATP depletion, have been reported to facilitate persister formation [49, 82]. Further in-depth studies on the mechanism of persister formation may assist academics in better understanding bacterial persisters and finding novel targets or compounds to treat persistent infections.

### 4.6. Limitations

The global field of bacterial persisters has rapidly expanded over the past two decades. To our knowledge, this study was the first bibliometric study reflecting the general trends of bacterial persisters. Compared to traditional reviews, this visualized study provided a large volume of information about the global distribution and trends of research on bacterial persisters. Moreover, like other bibliometric analyses, there were nonetheless several limitations of our study [83, 84]. Firstly, our data were retrieved from the WoSCC database and Ovid MEDLINE, but only publications in English were included. This potentially introduced language bias. Secondly, some recently published articles might have understated academic influence because of insufficient time to accumulate citations. The true contribution of these high-quality and recent publications in the field of bacterial persisters will become more apparent with time. Thirdly, the real contribution of authors in the cooperative network was difficult to distinguish as the software linked authors with the same weight or average weight. Authors with the greatest contribution could be provided by reading the original article. However, it was hard to decide the contribution of co-authors. Fourthly, even though the analysis is processed objectively by software, there are subjective biases to interpret the results.

## 5. Conclusions

In summary, this study presented the global trends in bacterial persisters based on bibliometric analysis. A series of studies were retrieved from the WoSCC and Ovid MEDLINE database, and 1,903 pieces of data were collected from 2001 to 2021. In total, there were 7,182 authors from 1,512 institutions in 74 countries who participated in the study of bacterial persisters. The USA was globally found as the leading country in this field, as it made the highest contribution to both the total number of publications and citation frequency. *Antimicrobial Agents and Chemotherapy* was the most influential journal with the great number of relevant publications, citations, and *h*-index. Kim Lewis achieved academic influence in the field of bacterial persisters with high publication volume and citation frequency. According to the bibliometric and visualized analyses, collaborations between researchers from different institutions should be strengthened to look deeper into bacterial persisters. In this study, we predicted that the publication outputs on bacterial persisters will continue with an upward trend in the coming decade. In-depth studies on the mechanism of persister formation will be the future research trend, and it will hopefully develop novel anti-persister methods to bring benefits to patients with persistent infections.

## Figures and Tables

**Table 1:** Annual growth rate (AGR), compound annual growth rate (CAGR), relative growth rate (RGR), and doubling time (DT) of the publications.

Year	Number of publication	Cumulative number of publications	AGR	CAGR	RGR	DT
2001	4	4		27.27%		
2002	8	12	100.00%		1.10	0.63
2003	5	17	-37.50%		0.35	1.99
2004	7	24	40.00%		0.34	2.01
2005	17	41	142.86%		0.54	1.29
2006	13	54	-23.53%		0.28	2.52
2007	17	71	30.77%		0.27	2.53

2008	21	92	23.53%	25.50%	0.26	2.67
2009	29	121	38.10%		0.27	2.53
2010	41	162	41.38%		0.29	2.37
2011	38	200	-7.32%		0.21	3.29
2012	59	259	55.26%		0.26	2.68
2013	80	339	35.59%		0.27	2.57
2014	93	432	16.25%		0.24	2.86
2015	103	535	10.75%		0.21	3.24

2016	158	693	53.40%	17.17%	0.26	2.68
2017	184	877	16.46%		0.24	2.94
2018	173	1,050	-5.98%		0.18	3.83
2019	210	1,260	21.39%		0.18	3.80
2020	294	1,554	40.00%		0.21	3.30
2021	349	1,903	18.71%		0.20	3.42

**Table 2:** The top 20 influential institutions on bacterial persisters (2001-2021).

Rank	Institution	Country	Publications	Citations	*h*-index
1	Northeastern University	USA	55	12,609	41
2	Harvard University	USA	44	6,043	39
3	Johns Hopkins University	USA	69	2,409	28
4	Pennsylvania State University	USA	49	2,841	28
5	University of Copenhagen	Denmark	62	4,767	24
6	Catholic University of Leuven	Belgium	54	2,677	23
7	Massachusetts General Hospital	USA	26	1,943	23
8	Princeton University	USA	28	2,023	20
9	Hebrew University of Jerusalem	Israel	25	5,367	20
10	Imperial College London	England	21	1,370	19
11	Harvard Medical School	USA	39	1,507	18
12	University of Tartu	Estonia	25	2,219	18
13	Brown University	USA	21	1,768	18
14	University of Florida	USA	41	1,482	17
15	Broad Institute of MIT and Harvard	USA	29	2,802	17
16	Texas A&M University	USA	24	1,846	17
17	Rutgers University	USA	25	808	16
18	Fudan University	China	28	634	14
19	Chinese Academy of Sciences	China	23	946	14
20	National University of Singapore	Singapore	23	627	14

**Table 3:** The top 19 productive authors on bacterial persisters (2001-2021).

Rank	Authors	Institutions	Publications	Citations
1	Ying Zhang	Johns Hopkins University	54	1,712
2	Kim Lewis	Northeastern University	49	12,295
3	Thomas K. Wood	Pennsylvania State University	48	3,072
4	Jan Michiels	Catholic University of Leuven	45	2,285
5	Maarten Fauvart	Catholic University of Leuven	28	1,529
6	Mark P. Brynildsen	Princeton University	24	2,217
7	Wanliang Shi	Johns Hopkins University	24	563
8	Kenn Gerdes	University of Copenhagen	23	3,126
9	Natalie Verstraeten	Catholic University of Leuven	19	859
10	Nathalie Q. Balaban	Hebrew University of Jerusalem	18	5,075
11	Jie Feng	Johns Hopkins University	18	391
12	Sophie Helaine	Imperial College London	18	2,136
13	Dacheng Ren	Syracuse University	18	362
14	Tanel Tenson	University of Tartu	18	1,676
15	Brian P. Conlon	University of North Carolina	17	1,475
16	James J. Collins	Broad Institute of MIT and Harvard	16	3,470
17	Mehmet A. Orman	University of Houston	16	762
18	Bram Van den Bergh	Catholic University of Leuven	16	853
19	Wenhong Zhang	Fudan University	15	316

**Table 4:** The top 10 popular journals publishing on bacterial persisters (2001-2021).

Rank	Journals	Publications	Citations	IFs^∗^	*h*-index
1	*Frontiers in Microbiology*	115	2,493	6.064	28
2	*Antimicrobial Agents and Chemotherapy*	113	5,725	5.938	41
3	*Journal of Bacteriology*	67	5,121	3.476	34
4	*mBio*	56	2,128	7.786	28
5	*PLOS One*	53	2,233	3.752	26
6	*Proceedings of the National Academy of Sciences*	50	3,465	12.779	32
7	*Scientific Reports*	48	1,197	4.996	21
8	*Methods in Molecular Biology (Clifton, N.J.)*	38	154	N/A	8
9	*Antibiotics-Basel*	35	649	5.222	11
10	*Journal of Antimicrobial Chemotherapy*	34	768	5.758	16

^∗^N/A was assigned when the journal impact factor was not available or had not been assigned in 2021.

## Data Availability

The data are available from the first author.

## Supplementary Materials

Supplementary Materials Supplementary Table 1: search strategies for bibliometric analysis. Supplementary Table 2: list of the top 20 productive institutions on bacterial persisters (2001-2021). Supplementary Table 3: list of the top 20 cited institutions on bacterial persisters (2001-2021). Supplementary Table 4: list of the top 10 cited journals publishing on bacterial persisters (2001-2021).
