# Supplementary material for: A Bibliometric Analysis of Research on Bacterial Persisters
Source: Biomed Res Int. 2023 Jan 6;2023:4302914. doi: 10.1155/2023/4302914 (PMC9839416; doi:10.1155/2023/4302914)
Supplement: Supplementary Materials — Supplementary Table 1: search strategies for bibliometric analysis. Supplementary Table 2: list of the top 20 productive institutions on bacterial persisters (2001-2021). Supplementary Table 3: list of the top 20 cited institutions on bacterial persisters (2001-2021). Supplementary Table 4: list of the top 10 cited journals publishing on bacterial persisters (2001-2021). [file 4302914.f1.docx]

Table S1. Search strategies for bibliometric analysis.

| Web of Science Core Collection | TS=(persister$ or antipersister$ or "antibiotic toleran*" or ((Escherichia or Staphylococcus or coli or aureus or Pseudomonas or Aemginosa or myobacteri* or bacteri* or microbial or antibiotic) near/5 persisten*))  Indexes=SCI-EXPANDED, SSCI, A&HCI, CPCI-S, CPCI-SSH, ESCI, CCR-EXPANDED; Timespan=2001-2021; Language=English; DocType="Article" OR "Review" |
| --- | --- |
| Ovid MEDLINE(R) <1946 to August Week 4 2022> | 1 (persister? or antipersister? or antibiotic toleran* or ((Escherichia or Staphylococcus or coli or aureus or Pseudomonas or Aemginosa or myobacteri* or bacteri* or microbial or antibiotic) adj5 persisten*)).mp. 7428  2 drug tolerance/ 21449  3 bacteria/ 170703  4 2 and 3 137  5 1 or 4 7529  6 limit 5 to (yr="2001 - 2021" and english and (journal article or "review")) (5597) |

Table S2 The top 20 productive institutions on bacterial persisters (2001-2021).

| Rank | Institutions | Countries | Publications | Citations | *h*-index |
| --- | --- | --- | --- | --- | --- |
| 1 | Johns Hopkins University | USA | 69 | 2,409 | 28 |
| 2 | University of Copenhagen | Denmark | 62 | 4,767 | 24 |
| 3 | Northeastern University | USA | 55 | 12,609 | 41 |
| 4 | Catholic University of Leuven | Belgium | 54 | 2,677 | 23 |
| 5 | Pennsylvania State University | USA | 49 | 2,841 | 28 |
| 6 | Harvard University | USA | 44 | 6,043 | 39 |
| 7 | University of Florida | USA | 41 | 1,482 | 17 |
| 8 | Harvard Medical School | USA | 39 | 1,507 | 18 |
| 9 | Russian Academy of Sciences | Russian | 33 | 407 | 10 |
| 10 | Broad Institute of MIT and Harvard | USA | 29 | 2,802 | 17 |
| 11 | Princeton University | USA | 28 | 2,023 | 20 |
| 12 | Fudan University | China | 28 | 634 | 14 |
| 13 | Massachusetts General Hospital | USA | 26 | 1,943 | 23 |
| 14 | Hebrew University of Jerusalem | Isreal | 25 | 5,367 | 20 |
| 15 | University of Tartu | Estonia | 25 | 2,219 | 18 |
| 16 | Texas A&M University | USA | 24 | 1,846 | 17 |
| 17 | Chinese Academy of Sciences | China | 23 | 946 | 14 |
| 18 | National University of Singapore | Singapore | 23 | 627 | 14 |
| 19 | University of Tuebingen | Germany | 22 | 723 | 14 |
| 20 | University of Basel | Switzerland | 22 | 981 | 12 |

Table S3 The top 20 cited institutions on bacterial persisters (2001-2021).

| Rank | Institutions | Countries | Publications | Citations | *h*-index |
| --- | --- | --- | --- | --- | --- |
| 1 | Northeastern University | USA | 55 | 12,609 | 41 |
| 2 | Harvard University | USA | 44 | 6,043 | 39 |
| 3 | Hebrew University of Jerusalem | Isreal | 25 | 5,367 | 20 |
| 4 | University of Copenhagen | Denmark | 62 | 4,767 | 24 |
| 5 | Rockefeller University | USA | 9 | 3,041 | 8 |
| 6 | Pennsylvania State University | USA | 49 | 2,841 | 28 |
| 7 | Broad Institute of MIT and Harvard | USA | 29 | 2,802 | 17 |
| 8 | Catholic University of Leuven | Belgium | 54 | 2,677 | 23 |
| 9 | Johns Hopkins University | USA | 69 | 2,409 | 28 |
| 10 | Boston University | USA | 10 | 2,308 | 10 |
| 11 | Montana State Univerdity | USA | 17 | 2,271 | 13 |
| 12 | University of Tartu | Estonia | 25 | 2,219 | 18 |
| 13 | Technical University of Denmark | Denmark | 16 | 2,211 | 12 |
| 14 | Rigshospitalet | Denmark | 12 | 2,181 | 10 |
| 15 | Princeton University | USA | 28 | 2,023 | 20 |
| 16 | Massachusetts General Hospital | USA | 26 | 1,943 | 23 |
| 17 | Texas A&M University | USA | 24 | 1,846 | 17 |
| 18 | Newcastle University | England | 12 | 1,789 | 11 |
| 19 | Brown University | USA | 21 | 1,768 | 18 |
| 20 | Harvard Medical School | USA | 39 | 1,507 | 18 |

Table S4 The top 10 cited journals publishing on bacterial persisters (2001-2021).

| Rank | Journals | Publications | Citations | IFs |
| --- | --- | --- | --- | --- |
| 1 | Antimicrobial Agents and Chemotherapy | 113 | 5,725 | 5.938 |
| 2 | Journal of Bacteriology | 67 | 5,121 | 3.476 |
| 3 | Science | 10 | 5,089 | 63.714 |
| 4 | Nature Reviews Microbiology | 13 | 4,970 | 78.297 |
| 5 | Proceedings of the National Academy of Sciences | 50 | 3,465 | 12.779 |
| 6 | Frontiers in Microbiology | 115 | 2,493 | 6.064 |
| 7 | Fems Microbiology Letters | 19 | 2,276 | 2.82 |
| 8 | Plos One | 53 | 2,233 | 3.752 |
| 9 | International Journal of Antimicrobial Agents | 6 | 2,175 | 15.441 |
| 10 | Mbio | 56 | 2,128 | 7.786 |
